# Supplementary material for: Dehydroepiandrosterone Sulfate, an Adrenal Androgen, Is Inversely Associated with Prevalence of Dynapenia in Male Individuals with Type 2 Diabetes
Source: Metabolites. 2023 Nov 3;13(11):1129. doi: 10.3390/metabo13111129 (PMC10673440; doi:10.3390/metabo13111129)
Supplement: Supplementary file 1 [file metabolites-13-01129-s001.zip › metabolites-2612850-supplementary.pdf]

**Table S1.**

Multiple linear regression analysis with identified variabales and medications used for determinants of SMI, HS and GS in individuals with T2D

| | SMI | | | HS | | | GS | | |
|---|---|---|---|---|---|---|---|---|---|
| Variables | t value | VIF | p value | t value | VIF | p value | t value | VIF | p value |
| Age | 9.127 | 1.554 | <0.001 | -6.768 | 1.782 | <0.001 | -5.200 | 1.563 | <0.001 |
| Male | 15.970 | 1.334 | <0.001 | 13.310 | 1.562 | <0.001 | -1.088 | 1.117 | 0.277 |
| BMI | 20.900 | 1.500 | <0.001 | 2.487 | 1.671 | 0.013 | -1.632 | 1.482 | 0.104 |
| Exercise (≥3Mets) | 2.704 | 1.138 | 0.004 | 2.0.32 | 1.114 | 0.043 | 2.747 | 1.140 | 0.006 |
| Duration of T2D | - | - | - | 1.478 | 1.733 | 0.141 | -0.484 | 1.238 | 0.628 |
| LDL-C | -4.000 | 1.320 | 0.007 | - | - | - | - | - | - |
| UA | - | - | - | -2.695 | 1.551 | 0.007 | - | - | - |
| Cr | 3.466 | 1.301 | <0.001 | 2.786 | 1.440 | 0.006 | - | - | - |
| ALB | - | - | - | 3.661 | 1.246 | <0.001 | 2.276 | 1.154 | 0.024 |
| DHEAS | - | - | - | 2.495 | 1.435 | 0.013 | - | - | - |
| ARB or ACEi | 0.799 | 1.355 | 0.425 | -0.390 | 1.409 | 0.697 | -0.587 | 1.371 | 0.588 |
| CCB | 0.723 | 1.444 | 0.470 | 0.487 | 1.402 | 0.627 | -0.476 | 1.090 | 0.635 |
| Statin | -3.841 | 1.274 | <0.001 | -0.044 | 1.197 | 0.965 | -0.208 | 1.140 | 0.835 |
| Antiplatelts | -1.467 | 1.435 | 0.143 | -2.051 | 1.266 | 0.041 | -4.002 | 1.208 | <0.001 |
| Su or Glinide | -1.218 | 1.263 | 0.224 | -1.832 | 1.326 | 0.178 | 0.251 | -1.266 | 0.802 |
| DPP4i | -0.080 | 1.548 | 0.936 | -0.850 | 1.578 | 0.396 | 1.479 | -1.540 | 0.140 |
| Metformin | 0.543 | 1.326 | 0.587 | 0.637 | 1.346 | 0.524 | 0.255 | 1.316 | 0.799 |
| $\alpha$ GI | 0.362 | 1.221 | 0.718 | -0.051 | 1.251 | 0.960 | 2.727 | -1.205 | <0.007 |
| Pioglitazone | 0.733 | 1.092 | 0.464 | -0.036 | 1.103 | 0.972 | 1.102 | 1.096 | 0.271 |
| SGLT2i | 0.317 | 1.191 | 0.751 | 0.847 | 1.327 | 0.397 | 0.641 | 1.185 | 0.522 |
| Insulin | -0.843 | 1.184 | 0.400 | -0.495 | 1.330 | 0.621 | 0.873 | -1.195 | 0.383 |
| GLP-1RA | 1.446 | 1.474 | 0.149 | 1.350 | 1.506 | 0.178 | 1.598 | -1.473 | 0.111 |

**Table S2.**

Multiple logistic regression analysis including GNRI for determinants of dynapenia in male and female individuals with T2D

| | Dynapenia | | | | | | | |
|---|---|---|---|---|---|---|---|---|
| | Males | | | | Females | | | |
| Variables | OR | 95% CI | VIF | p value | OR | 95% CI | VIF | p value |
| Age (1year) | 1.136 | 1.057 to 1.236 | 2.121 | 0.001 | 1.265 | 1.150 to 1.424 | 1.549 | <0.001 |
| BMI (1kg/m$^2$) | 1.144 | 0.947 to 1.398 | 3.776 | 0.169 | 0.928 | 0.730 to 1.130 | 3.076 | 0.481 |
| Exercise (≥3Mets) | 0.294 | 0.100 to 0.793 | 1.105 | 0.020 | 0.943 | 0.247 to 3.364 | 1.190 | 0.929 |
| Current Smoking | 1.306 | 0.318 to 5.230 | 1.296 | 0.705 | 4.362 | 0.152 to 57.30 | 1.096 | 0.296 |
| Hypertension | 1.421 | 0.464 to 4.399 | 1.560 | 0.537 | 0.987 | 0.251 to 3.886 | 1.480 | 0.985 |
| Dyslipidemia | 0.305 | 0.078 to 1.116 | 1.271 | 0.078 | 1.998 | 0.459 to 10.43 | 1.199 | 0.379 |
| Duration of T2D (1year) | 1.061 | 1.003 to 1.126 | 1.276 | 0.045 | 0.984 | 0.929 to 1.037 | 1.316 | 0.556 |
| SBP (1mmHg) | 0.975 | 0.940 to 1.008 | 1.469 | 0.141 | 1.015 | 0.983 to 1.040 | 1.337 | 0.275 |
| LDL-C (1mg/dL) | 0.997 | 0.979 to 1.016 | 1.348 | 0.763 | 0.992 | 0.970 to 1.013 | 1.272 | 0.441 |
| HDL-C (1mg/dL) | 1.007 | 0.962 to 1.051 | 1.380 | 0.807 | 0.994 | 0.948 to 1.041 | 1.545 | 0.810 |
| TG (1mg/dL) | 1.005 | 0.998 to 1.014 | 1.464 | 0.192 | 0.995 | 0.984 to 1.006 | 1.548 | 0.384 |
| HbA1c (1%) | 0.845 | 0.547 to 1.176 | 1.351 | 0.358 | 1.070 | 0.701 to 1.517 | 1.238 | 0.727 |
| UA (1mg/dL) | 1.505 | 0.939 to 2.522 | 1.319 | 0.102 | 0.789 | 0.439 to 1.373 | 1.690 | 0.410 |
| Cr (1mg/dL) | 0.727 | 0.078 to 2.431 | 1.228 | 0.715 | 0.078 | 0.002 to 1.968 | 1.543 | 0.138 |
| GNRI (1 index) | 0.936 | 0.852 to 1.116 | 3.985 | 0.133 | 1.035 | 0.967 to 1.144 | 3.122 | 0.379 |
| DHEAS (1μg/dL) | 0.985 | 0.975 to 0.993 | 1.372 | 0.001 | 1.007 | 0.999 to 1.015 | 1.293 | 0.076 |
